# Cardiovascular Events with the Use of Long-Acting Muscarinic Receptor Antagonists: An Analysis of the FAERS Database 2020–2023

**DOI:** 10.1007/s00408-024-00677-3

**Published:** 2024-02-06

**Authors:** Maria Gabriella Matera, Luigino Calzetta, Paola Rogliani, Nicola Hanania, Mario Cazzola

**Affiliations:** 1 Unit of Pharmacology, Department of Experimental Medicine, School of Medicine, University of Campania “Luigi Vanvitelli”, Naples, Italy; 2 Unit of Respiratory Disease and Lung Function, Department of Medicine and Surgery, University of Parma, Parma, Italy; 3 Unit of Respiratory Medicine, Department Experimental Medicine, University of Rome “Tor Vergata”, Rome, Italy; 4 Section of Pulmonary and Critical Care Medicine, Baylor College of Medicine, Houston, TX USA

**Keywords:** Cardiovascular adverse events, Dual bronchodilation, FAERS, LAMAs, Triple therapy

## Abstract

**Purpose:**

This study aimed to examine reports of cardiovascular adverse events (CV AEs) observed in the real-world during treatment with aclidinium, tiotropium, glycopyrronium, and umeclidinium alone or in combination with a LABA and, in the context of triple therapy, with the addition of an ICS, and submitted to the food and drug administration adverse event reporting system (FAERS).

**Methods:**

A retrospective disproportionality analysis was conducted utilizing CV AE reports submitted to the FAERS from January 2020 to 30 September 2023. Disproportionality was measured by calculating the reporting odds ratio.

**Results:**

Compared with ipratropium, tiotropium was associated with fewer reports of CV AEs. Compared with tiotropium, other LAMAs were more likely to be associated with reports of CV AEs. Combinations of glycopyrronium with indacaterol or formoterol and umeclidinium with vilanterol significantly reduced reports of CV AEs compared with the respective LAMA. The addition of an ICS to these combinations further reduced the risk of CV AE reports.

**Conclusion:**

Our study suggests that inhaled LAMAs are not free from cardiac AE risks. This risk may be more evident when the newer LAMAs are used, but it is generally significantly reduced when COPD patients are treated with dual bronchodilators or triple therapy. However, these results do not prove that LAMAs cause CV AEs, as FAERS data alone are not indicative of a drug’s safety profile. Given the frequency with which COPD and cardiovascular disease co-exist, a large study in the general population could shed light on this very important issue.

## Introduction

Long-acting muscarinic receptor (mAChR) antagonists (LAMAs), given as monotherapy or in combination with a long-acting β_2_-agonist (LABA) and, often, an inhaled corticosteroid (ICS), are widely prescribed for the treatment of chronic obstructive pulmonary disease (COPD) [1]. However, there are concerns about possible associations between their use and cardiovascular disease (CVD) [2, 3].

One observational study using health administrative data from Ontario (Canada) reported a significantly increased CV risk associated with the new use of LAMAs compared with to non-use of this class of bronchodilators [4]. However, according to a systematic review and meta-analysis of the currently available literature on their CV adverse events (AEs), LAMAs appear to be safe even in patients with heart disease, compared with other active drugs or a placebo [5].

Although pivotal clinical trials have provided much of the current information, the AE rates seen in such trials may not reflect those seen in real-world practice [6]. Therefore, real-world studies are needed to identify high-risk individuals who may benefit from electrocardiogram (ECG) monitoring [3], particularly as the CV response to mAChR blockade may vary between patients and with underlying comorbidities [7].

Four LAMAs are approved for the treatment of COPD: aclidinium bromide, tiotropium bromide, glycopyrronium bromide, and umeclidinium bromide. While the pharmacological profiles of these LAMAs differ, all four have a longer residence time at the M_3_ mAChR, whose blockade causes airway smooth muscle relaxation, and a shorter residence time at the M_2_ mAChR compared to short-acting mAChR antagonists (SAMAs), such as ipratropium bromide, which have non-selective binding properties [8]. Differences in the pharmacological properties of these LAMAs suggest that there may be different effects on the heart depending on the agent used. However, no clinical trials have addressed this issue, and there is still a lack of general analysis of the potential CV risk of different LAMAs.

In this study, we reviewed data from the US Food and Drug Administration’s (FDA) adverse event reporting system (FAERS) to examine reports of CV AEs observed in the real-world during treatment with aclidinium, tiotropium, glycopyrronium, and umeclidinium alone or in combination with a LABA and, in the context of triple therapy, with the addition of an ICS.

## Materials and Methods

The FAERS is a centralized, computerized information database that collects spontaneous reports of AEs associated with the administration of drugs and therapeutic biologics submitted directly by healthcare personnel and consumers or indirectly through manufacturers from the US and other countries using MedWATCH program submission forms [9]. It provides a helpful insight into the AE profile of drugs because of the broad exposure to a given drug in the real-world population and the large sample size with a wide range of AE reports [10].

The FAERS codes AEs based on the preferred term (PT) level of the standardized terminology of the medical dictionary for regulatory activities (MedDRA) Version 26.1. In the present analysis, AEs coded by PTs belonging to the MedDRA system organ classes “cardiac disorder’” were considered outcomes of interest. A narrower version examined standardized MedDRA queries. We assessed “cardiac arrhythmias,” “cardiac failure,” and “ischemic heart disease” because there is evidence that inhaled mAChR antagonists induce pro-arrhythmic and pro-ischemic effects [11], and there is also some, although inconsistent, evidence of an increased risk of heart failure (HF) with the use of these drugs [12]. We were aware that each report could include one or more CV AEs, as highlighted by FEARS.

Disproportionality in pharmacovigilance occurs when a drug is associated with an AE [13]. Since only odds ratios can be obtained for studies in which it is typically not possible to estimate the population at risk [14], as in the case of our investigation, we used the reported odds ratio (ROR) to find statistical associations between CV AEs and LAMAs. The ROR is a disproportionate measure based on the ratio of the odds of cases in reports for a given drug to the odds in reports where that drug is not present in the FAERS database [15]. A 95% confidence interval (CI) lower limit greater than one was considered a statistically significant ROR if there were at least 3 cases [16].

For the scope of the present study, we considered reports submitted to the FAERS database from January 2020 to 30 September 2023. All reports with generic LAMA names (aclidinium bromide, glycopyrrolate, glycopyrronium, tiotropium, tiotropium bromide, tiotropium bromide anhydrous, tiotropium bromide monohydrate, umeclidinium, umeclidinium bromide) were extracted. As a significant effect of exposure to ipratropium bromide on the risk of CV AEs has been reported [17], we also included data on this relatively non-selective SAMA (ipratropium, ipratropium bromide, ipratropium bromide anhydrous) in the analysis for comparison with tiotropium. Tiotropium was the first LAMA to enter daily use and remains the most prescribed compound in this class of bronchodilators, at least in the US [18].

As data in the FAERS database are anonymized, ethics committee approval was not required for this analysis.

## Results

We examined 18,208 reports of AEs that occurred during treatment with a LAMA, with the majority (12,472) attributed to tiotropium. There were more AEs in females than in males (9832 vs. 6231), at least in cases where sex was reported. The highest prevalence of AEs was in the age group 65–85 years (5771 reports), followed by the age group younger than 65 years (5054 reports).

There were 2261 CV AEs reported (12.4% of the total), with a higher number of males (1055) than females (940). The age group 65–85 years also had the highest number of reports of CV AEs (941), followed by the age group younger than 65 years (702 reports).

Compared to ipratropium, tiotropium was associated with fewer reports of CV AEs [ROR 0.53 (0.48–0.58)].

Table 1 shows the number of reported cases of cardiac disorders with each LAMA, then subdivided by sex and age of patients.

**Table 1:** Number of reported cases of cardiac disorders with each LAMA, then subdivided by sex and age of patients

LAMA	Number of AE cases	Number of CD cases	Female	Male	NS	Less than18 years	18–64 years	65–85 years	More than 85 years	NS
Tiotropium	12,472	1238	502	599	137		363	564	48	164
Aclidinium	1254	188	86	74	28		54	77	19	38
Glycopyrronium	1883	376	207	128	41	22	180	98	18	60
Umeclidinium	2599	459	145	254	60		105	202	52	100

*AE* adverse event, *CD* cardiac disorder, *NS* not specified

**Fig. 1:**
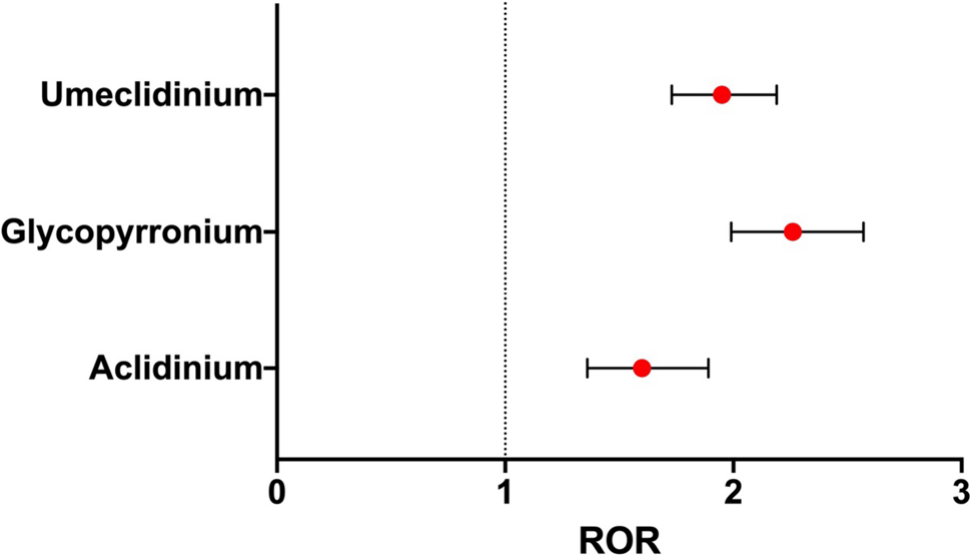
Forest plots of disproportionality [reported odds ratio (ROR)] of LAMAs and cardiovascular events compared with tiotropium

Compared to tiotropium, other LAMAs were more likely to be associated with reports of CV AEs. In this case, the ROR was the ratio of the odds of the number of CV events associated with tiotropium to those associated with other LAMAs [aclidinium: ROR 1.60 (1.36–1.89); glycopyrronium: ROR 2.26 (1.99–2.57); umeclidinium: ROR 1.95 (1.73–2.19)] (Fig. 1).

**Fig. 2:**
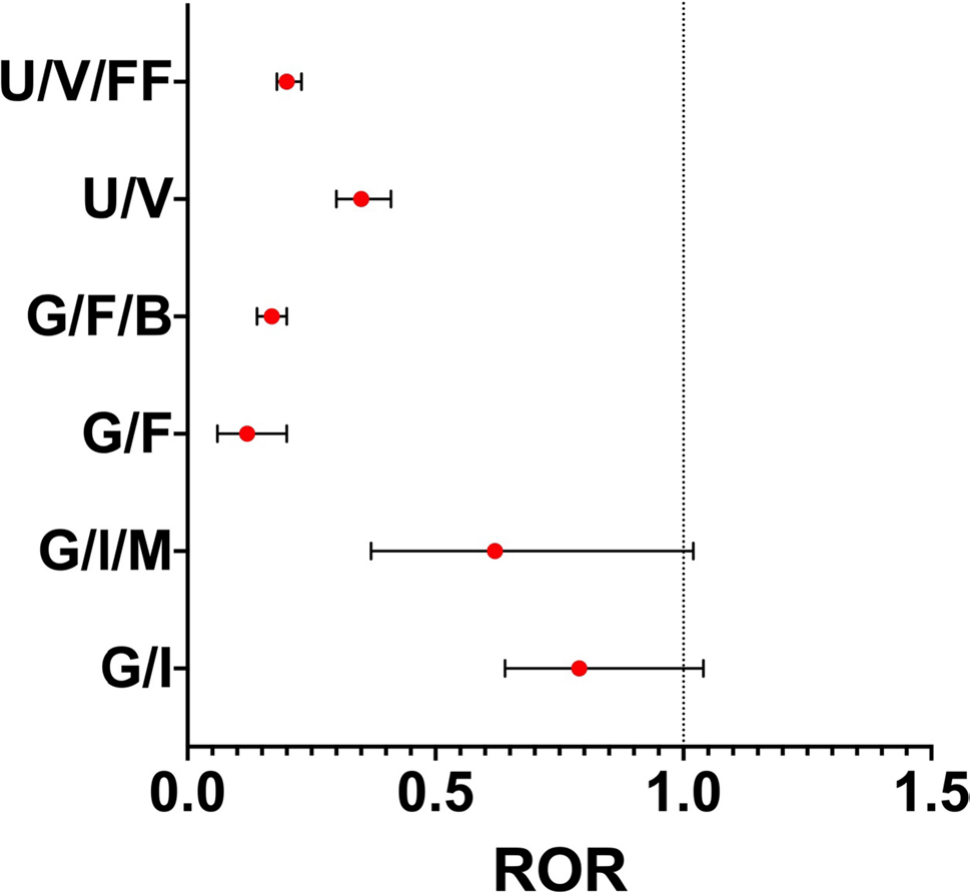
Forest plots of disproportionality [reported odds ratio (ROR)] of dual bronchodilation and triple therapy including glycopyrronium or umeclidinium, respectively, and cardiovascular events compared with glycopyrronium or umeclidinium. *F* formoterol, *FF* fluticasone furoate, *G* glycopyrronium, *I* indacaterol, *M* mometasone, *U* umeclidinium, *V* vilanterol

As glycopyrronium and umeclidinium were developed with a LABA and as part of a triple therapy with an ICS, we calculated the ROR of dual bronchodilators and triple combinations compared with LAMA monotherapy (Fig. 2). Compared with glycopyrronium monotherapy, glycopyrronium/indacaterol and glycopyrronium/indacaterol/mometasone were associated with significantly fewer reports of CV AEs, with RORs of 0.79 [0.64–0.97] and 0.62 [0.37–1.02], respectively. This was also the case when formoterol was added to glycopyrronium, with a ROR compared to glycopyrronium monotherapy of < 1 [0.12 (0.06–0.20)]. The addition of budesonide did not significantly change this ROR [0.17 (0.14–0.20)]. Similarly, umeclidinium/vilanterol and umeclidinium/vilanterol/fluticasone furoate were associated with significantly fewer reports of CV AEs compared to umeclidinium alone [ROR 0.35 (0.30–0.41) and 0.20 (0.18–0.23), respectively].

Table 2 describes the impact of each LAMA on the three main subgroups of CV AEs (arrhythmia, HF, and ischemic heart disease). Compared to all reports of CV AEs in the three groups considered, aclidinium had the lowest percentage of events assigned to the arrhythmia group. In contrast, more than 50% of the reports of CV AEs during treatment with umeclidinium were assigned to this group. The opposite was true when looking at events in the ischemic heart disease subgroup, with umeclidinium having the lowest and aclidinium the highest percentage compared to the total number of CV AEs reported.

**Table 2:** Details on the specific cardiac events divided into three major subgroups (arrhythmias, heart failure, and ischemic heart disease) and by LAMA

Subgroup of cardiac AEs	Tiotropium	%	Aclidinium	%	Glycopyrronium	%	Umeclidinium	%
Arrhythmias	373	37.3	52	25.1	187	48.2	194	51.4
Heart failure	211	21.1	24	11.6	40	10.3	92	24.3
Ischemic heart disease	417	41.6	131	63.3	161	41.5	92	24.3

% percent of reported cases of cardiac disorders for that LAMA

## Discussion

The results of this retrospective pharmacovigilance analysis documented that the use of tiotropium significantly reduced the reporting of CV AEs compared with ipratropium while the other three LAMAs (aclidinium, glycopyrrolate, and umeclidinium), which were introduced into clinical use after tiotropium, were found to be associated with more reported cardiac AEs than tiotropium, although the available evidence from randomized controlled trials (RCTs) generally emphasizes the CV safety of these LAMA.

The glycopyrronium data were completely unexpected. It is the only mAChR antagonist to date to have a greater relative affinity for M_3_ than M_2_ mAChRs [19]. Glycopyrronium had a lower M_2_ mAChR occupancy and a safer CV profile than tiotropium in an integrated rat model [20]. The percentage of patients with new or worsened clinically significant QTcF values was slightly higher with tiotropium (5.8%) than with glycopyrronium (4.0%) when these two LAMAs were compared in the GLOW5 clinical trial [21]. However, two patients on glycopyrronium had QTcF values > 480 ms compared with none on tiotropium and the percentage of patients with an increase in QTcF of 30–60 ms from baseline was slightly higher in the glycopyrronium group (3.4% vs. 3.0%). Furthermore, in a recent real-world study on the safety and efficacy of glycopyrronium in Japanese patients with COPD, the incidence of cardiac AEs was 2.98% [22]. The common AEs were HF in 0.55%, myocardial infarction in 0.39%, angina pectoris and ventricular extrasystoles in 0.31% of patients each.

Aclidinium also exhibits M_3_ mAChRs versus M_2_ mAChRs selectivity and, unlike other LAMAs, is rapidly hydrolyzed in human plasma into an acid and an alcohol metabolite, neither of which binds to mAChRs [23]. A post-approval safety study, conducted in the United Kingdom using de-identified data from primary care practices, showed that the crude incidence rates per 1000 person-years of acute myocardial infarction during current use were 10.29 for aclidinium, 11.33 for tiotropium, and 11.86 for other LAMAs, while those for major adverse cardiac events (MACE) were 16.00, 17.98, and 18.18, respectively [24]. In addition, a North American RCT proved that aclidinium was non-inferior to placebo for the risk of MACE in patients with COPD and increased CV risk over 3 years [25].

Umeclidinium exhibits kinetic selectivity for M_3_ over M_2_ mAChRs and dissociates more slowly from M_3_ mAChRs than from M_2_ mAChRs (half-lives: 82 and 9 min, respectively) [26]. Its dissociation from M_2_ and M_3_ mAChRs is faster than that of tiotropium (approximately four and three times faster, respectively). Studies using monotherapy for more than four weeks showed that both umeclidinium 62.5 μg and 125 μg caused cardiac-related AEs compared to placebo [27]. Supraventricular tachycardia, atrial ectopy, and atrial fibrillation were the most common AEs. However, there was no evidence of a higher risk of significant MACEs with either doses of umeclidinium compared to placebo. On the other hand, umeclidinium was associated with a higher incidence of several CV events and post-baseline ECG abnormalities in subjects with underlying CV risk factors compared to placebo [28]. However, to the best of our knowledge, there is no direct or indirect comparison between umeclidinium monotherapy and tiotropium monotherapy in terms of CV risk.

It is difficult to explain the discrepancy between what has been reported in the literature and what was observed in this study. It has been suggested that there may be a variable CV response to mAChR antagonism in individual patients [29]. Regulator of G protein signaling 6 (RGS6), which accelerates the deactivation kinetics of the G protein-gated K^+^ channel (I_KACh_) in sinoatrial node cells and atrial myocytes, thereby limiting parasympathetic activation to avoid parasympathetic override and severe bradycardia, controls the duration of G protein activation in the heart [30]. Altered parasympathetic signaling through the M_2_ mAChR-I_KACh_ pathway may affect ventricular electrophysiological properties distinct from its influence on atrial physiology [31]. A genetic predisposition associated with a modification of RGS6 may influence the onset of CV AEs [32]. The chance of treating patients who are particularly sensitive to M_2_ mAChR blockade is certainly greater in an unselected general population. However, this does not explain the difference between tiotropium and the other LAMAs.

Cell-to-cell communication and ACh-induced endothelium-dependent dilation of coronary arteries are mediated by activation of M_3_ mAChRs present in cardiac cells [33], which also stimulates the delayed rectifier potassium current, thereby regulating cardiac rhythm and repolarization and exerting cytoprotective effects against myocardial damage, including ischemia [34, 35]. The function of these mAChRs may become prominent in pathological conditions such as cardiac ischemia, pathological cardiac hypertrophy, arrhythmia, and HF [34]. Blocking M_3_ mAChRs with mAChR antagonism abolishes this protective effect.

When cardiac M_3_ mAChR activity is increased, a greater M_3_/M_2_ mAChR selectivity may be an issue. Aclidinium [36], glycopyrrolate [37], and umeclidinium [26] have higher M_3_/M_2_ mAChR selectivity than tiotropium, which may explain why more CV AEs have been reported in users of these three LAMAs. However, lack of specific information on pathological conditions of patients with reported cardiac disorders prevents confirmation of this hypothesis. Given the frequency with which COPD and CVD co-exist, an appropriate study could shed light on this issue that is of great importance [29, 38].

Not surprisingly, the addition of a LABA to a LAMA reduces the likelihood of reported cardiac events. Although there are concerns about the possible association between the use of dual bronchodilators and CV morbidity in patients with COPD, as LABAs also have a high potential to influence cardiac activity [6], the COPD and Systemic Consequences-Comorbidities Network (COSYCONET) observational study documented the beneficial effect of dual bronchodilation on the CV system [39]. In addition, a systematic review with meta-analysis that included data from trials of at least 3 months duration showed that umeclidinium/vilanterol appeared to provide a protective signal against cardiac AEs, whereas glycopyrrolate/indacaterol significantly protected against such events compared with mono-components [40]. However, the data from these studies were mainly derived from patients selected for enrollment in RCTs, who generally do not have severe CV morbidity.

A likely explanation for the reduced risk of cardiac AEs with dual bronchodilation is related to the deflating effect of this therapy, with less compression of the pulmonary microcirculation and increasing perfusion [41]. In patients with moderate to severe COPD and pulmonary hyperinflation, a 14-day course of LAMA + LABA resulted in significant lung deflation, normalized biventricular end-diastolic volumes, and improved cardiac filling [42]. Another closely related mechanism is improved regional ventilation due to bronchodilation [43]. An increase in regional ventilation could improve the ventilation-perfusion mismatch, thereby improving the venous blood flow in the left heart.

Adding an ICS to glycopyrronium/indacaterol and umeclidinium/vilanterol extended the significant reduction in the risk of CV AEs observed with dual bronchodilation versus LAMA alone. This is not surprising, as a recent random-effects meta-analysis documented an association between ICS-containing medications and reduced CV risk in COPD patients [44]. In any case, it should be mentioned that glycopyrronium/indacaterol/mometasone has only been approved for asthma treatment.

While the results of this research are certainly interesting, they do not document that LAMAs cause CV AEs [9]. The reliance on FAERS data alone is not sufficient to determine the safety profile of a drug [9]. In fact, stablishing a cause-and-effect relationship between a drug and an AE is not possible with FAERS data because the total number of patients using the drug is not available in the database. Consequently, the incidence of AEs cannot be accurately calculated and only a rough estimate based on the signal strength (ROR value) is possible. Furthermore, it is important to recognize that our analysis is subject to inherent bias due to the FAERS spontaneous reporting mechanism. AE reports are voluntarily submitted by healthcare providers, consumers, and manufacturers. Therefore, they may contain false, exaggerated, inaccurate, incomplete, and delayed information. In addition, these reports often lack medical review, increasing the likelihood of misclassification of cases. In our study, the fact that most reports concerned tiotropium and that it was not possible to adjust for underlying comorbidities and duration of treatment were other limitations.

Nevertheless, despite these limitations, our study suggests that inhaled LAMAs are not free from cardiac AE risks. This risk may be more evident when the newer LAMAs are used but it is generally significantly reduced when COPD patients are treated with dual bronchodilators or triple therapy.

## References

[1] Bloom CI, Montonen J, Jöns O, Garry EM, Bhatt SP (2022). First maintenance therapy for chronic obstructive pulmonary disease: retrospective analyses of US and UK healthcare databases. Pulm Ther.

[2] Cazzola M, Page C, Matera MG (2013). Long-acting muscarinic receptor antagonists for the treatment of respiratory disease. Pulm Pharmacol Ther.

[3] Stolz D, Cazzola M, Martínez-García MA, Pépin J-L, Cazzola M (2020). Characterising the cardiovascular safety profile of inhaled muscarinic receptor antagonists. Cardiovascular complications of respiratory disorders (ERS monograph).

[4] Gershon A, Croxford R, Calzavara A, To T, Stanbrook MB, Upshur R, Stukel TA (2013). Cardiovascular safety of inhaled long-acting bronchodilators in individuals with chronic obstructive pulmonary disease. JAMA Intern Med.

[5] Zhang C, Zhang M, Wang Y, Xiong H, Huang Q, Shuai T, Liu J (2021). Efficacy and cardiovascular safety of LAMA in patients with COPD: a systematic review and meta-analysis. J Investig Med.

[6] Matera MG, Rogliani P, Calzetta L, Cazzola M (2016). Safety considerations with dual bronchodilator therapy in COPD: an update. Drug Saf.

[7] Cazzola M, Calzetta L, Rogliani P, Matera MG (2017). Tiotropium formulations and safety: a network meta-analysis. Ther Adv Drug Saf.

[8] Matera MG, Belardo C, Rinaldi M, Rinaldi B, Cazzola M (2020). Emerging muscarinic receptor antagonists for the treatment of asthma. Expert Opin Emerg Drugs.

[9] U.S. Food and Drug Administration (2023) FDA adverse event reporting system (FAERS) public dashboard. https://www.fda.gov/drugs/questions-and-answers-fdas-adverse-event-reporting-system-faers/fda-adverse-event-reporting-system-faers-public-dashboard. Accessed 15 Dec 2023

[10] Silberstein SD, Reshef S, Cohen JM, Gandhi S, Seminerio M, Ramirez Campos V, Kessler Y, Thompson SF, Blumenfeld A (2023). Adverse events reported with therapies targeting the CGRP pathway during the first 6 months post-launch: a retrospective analysis using the FDA adverse events reporting system. Adv Ther.

[11] Singh S, Loke YK, Enright P, Furberg CD (2013). Pro-arrhythmic and pro-ischaemic effects of inhaled anticholinergic medications. Thorax.

[12] Verhamme KM, Afonso AS, van Noord C, Haag MD, Koudstaal PJ, Brusselle GG, Sturkenboom MC (2012). Tiotropium Handihaler and the risk of cardio- or cerebrovascular events and mortality in patients with COPD. Pulm Pharmacol Ther.

[13] Caster O, Aoki Y, Gattepaille LM, Grundmark B (2020). Disproportionality analysis for pharmacovigilance signal detection in small databases or subsets: recommendations for limiting false-positive associations. Drug Saf.

[14] Sedgwick P (2013). Case-control studies: measures of risk. BMJ.

[15] Rothman KJ, Lanes S, Sacks ST (2004). The reporting odds ratio and its advantages over the proportional reporting ratio. Pharmacoepidemiol Drug Saf.

[16] Zhai Y, Ye X, Hu F, Xu J, Guo X, Zhuang Y, He J (2019). Endocrine toxicity of immune checkpoint inhibitors: a real-world study leveraging US Food and drug administration adverse events reporting system. J Immunother Cancer.

[17] Ogale SS, Lee TA, Au DH, Boudreau DM, Sullivan SD (2010). Cardiovascular events associated with ipratropium bromide in COPD. Chest.

[18] ClinCalc.com (2023) Tiotropium. Drug usage statistics, United States, 2013–2020. https://clincalc.com/DrugStats/Drugs/Tiotropium. Accessed 18 Dec 2023

[19] Tashkin DP, Gross NJ (2018). Inhaled glycopyrrolate for the treatment of chronic obstructive pulmonary disease. Int J Chron Obstruct Pulmon Dis.

[20] Trifilieff A, Ethell BT, Sykes DA, Watson KJ, Collingwood S, Charlton SJ, Kent TC (2015). Comparing the cardiovascular therapeutic indices of glycopyrronium and tiotropium in an integrated rat pharmacokinetic, pharmacodynamic and safety model. Toxicol Appl Pharmacol.

[21] Chapman KR, Beeh KM, Beier J, Bateman ED, D'Urzo A, Nutbrown R, Henley M, Chen H, Overend T, D'Andrea P (2014). A blinded evaluation of the efficacy and safety of glycopyrronium, a once-daily long-acting muscarinic antagonist, versus tiotropium, in patients with COPD: the GLOW5 study. BMC Pulm Med.

[22] Kato C, Wang D, Nakamura N, Sasajima T, Yoshisue H (2022). Real-world safety and efficacy of glycopyrronium bromide in Japanese patients with COPD: a 52-week post-marketing surveillance. Open Respir Med J.

[23] Gavaldà A, Ramos I, Carcasona C, Calama E, Otal R, Montero JL, Sentellas S, Aparici M, Vilella D, Alberti J, Beleta J, Miralpeix M (2014). The in vitro and in vivo profile of aclidinium bromide in comparison with glycopyrronium bromide. Pulm Pharmacol Ther.

[24] Rebordosa C, Plana E, Rubino A, Aguado J, Martinez D, Lei A, Daoud S, Saigi-Morgui N, Perez-Gutthann S, Rivero-Ferrer E (2022). Risk assessment of acute myocardial infarction and stroke associated with long-acting muscarinic antagonists, alone or in combination, versus long-acting beta2-agonists. Int J Chron Obstruct Pulmon Dis.

[25] Wise RA, Chapman KR, Scirica BM, Bhatt DL, Daoud SZ, Zetterstrand S, Reisner C, Gil EG (2019). Effect of aclidinium bromide on major cardiovascular events and exacerbations in high-risk patients with chronic obstructive pulmonary disease: the ASCENT-COPD randomized clinical trial. JAMA.

[26] Salmon M, Luttmann MA, Foley JJ, Buckley PT, Schmidt DB, Burman M, Webb EF, DeHaas CJ, Kotzer CJ, Barrett VJ, Slack RJ, Sarau HM, Palovich MR, Lainé DI, Hay DW, Rumsey WL (2013). Pharmacological characterization of GSK573719 (umeclidinium): a novel, long-acting, inhaled antagonist of the muscarinic cholinergic receptors for treatment of pulmonary diseases. J Pharmacol Exp Ther.

[27] Babu KS, Morjaria JB (2017). Umeclidinium in chronic obstructive pulmonary disease: latest evidence and place in therapy. Ther Adv Chronic Dis.

[28] Donohue JF, Niewoehner D, Brooks J, O'Dell D, Church A (2014). Safety and tolerability of once-daily umeclidinium/vilanterol 125/25 mcg and umeclidinium 125 mcg in patients with chronic obstructive pulmonary disease: results from a 52-week, randomized, double-blind, placebo-controlled study. Respir Res.

[29] Cazzola M, Calzetta L, Rinaldi B, Page C, Rosano G, Rogliani P, Matera MG (2017). Management of chronic obstructive pulmonary disease in patients with cardiovascular diseases. Drugs.

[30] Posokhova E, Ng D, Opel A, Masuho I, Tinker A, Biesecker LG, Wickman K, Martemyanov KA (2013). Essential role of the m2R-RGS6-IKACh pathway in controlling intrinsic heart rate variability. PLoS ONE.

[31] Kulkarni K, Xie X, Fernandez M, de Velasco E, Anderson A, Martemyanov KA, Wickman K, Tolkacheva EG (2018). The influences of the M2R-GIRK4-RGS6 dependent parasympathetic pathway on electrophysiological properties of the mouse heart. PLoS ONE.

[32] Rorabaugh BR, Chakravarti B, Mabe NW, Seeley SL, Bui AD, Yang J, Watts SW, Neubig RR, Fisher RA (2017). Regulator of G protein signaling 6 protects the heart from ischemic injury. J Pharmacol Exp Ther.

[33] Patanè S (2014). M3 muscarinic acetylcholine receptor in cardiology and oncology. Int J Cardiol.

[34] Hang P, Zhao J, Qi J, Wang Y, Wu J, Du Z (2013). Novel insights into the pervasive role of M_3_ muscarinic receptor in cardiac diseases. Curr Drug Targets.

[35] Saternos HC, Almarghalani DA, Gibson HM, Meqdad MA, Antypas RB, Lingireddy A, AbouAlaiwi WA (2018). Distribution and function of the muscarinic receptor subtypes in the cardiovascular system. Physiol Genom.

[36] Gavaldà A, Miralpeix M, Ramos I, Otal R, Carreño C, Viñals M, Doménech T, Carcasona C, Reyes B, Vilella D, Gras J, Cortijo J, Morcillo E, Llenas J, Ryder H, Beleta J (2009). Characterization of aclidinium bromide, a novel inhaled muscarinic antagonist, with long duration of action and a favorable pharmacological profile. J Pharmacol Exp Ther.

[37] Buhl R, Banerji D (2012). Profile of glycopyrronium for once-daily treatment of moderate-to-severe COPD. Int J Chron Obstruct Pulmon Dis.

[38] Cazzola M, Rogliani P, Matera MG (2015). Cardiovascular disease in patients with COPD. Lancet Respir Med.

[39] Kellerer C, Kahnert K, Trudzinski FC, Lutter J, Berschneider K, Speicher T, Watz H, Bals R, Welte T, Vogelmeier CF, Jörres RA, Alter P (2021). COPD maintenance medication is linked to left atrial size: results from the COSYCONET cohort. Respir Med.

[40] Calzetta L, Rogliani P, Matera MG, Cazzola M (2016). A systematic review with meta-analysis of dual bronchodilation with LAMA/LABA for the treatment of stable COPD. Chest.

[41] Cazzola M, Page C, Rogliani P, Calzetta L, Matera MG (2022). Dual bronchodilation for the treatment of COPD: from bench to bedside. Br J Clin Pharmacol.

[42] Hohlfeld JM, Vogel-Claussen J, Biller H, Berliner D, Berschneider K, Tillmann HC, Hiltl S, Bauersachs J, Welte T (2018). Effect of lung deflation with indacaterol plus glycopyrronium on ventricular filling in patients with hyperinflation and COPD (CLAIM): a double-blind, randomised, crossover, placebo-controlled, single-centre trial. Lancet Respir Med.

[43] Vogel-Claussen J, Schönfeld CO, Kaireit TF, Voskrebenzev A, Czerner CP, Renne J, Tillmann HC, Berschneider K, Hiltl S, Bauersachs J, Welte T, Hohlfeld JM (2019). Effect of indacaterol/glycopyrronium on pulmonary perfusion and ventilation in hyperinflated patients with chronic obstructive pulmonary disease (CLAIM): a double-blind, randomized crossover trial. Am J Respir Crit Care Med.

[44] Gadhvi K, Kandeil M, Raveendran D, Choi J, Davies N, Nanchahal S, Wing O, Quint J, Whittaker H (2023). Inhaled corticosteroids and risk of cardiovascular disease in chronic obstructive pulmonary disease: a systematic review and meta-regression. Chronic Obstr Pulm Dis.

